# S6 Peptide Derived from KvAP Channel Shows Cooperativity in Gating on Bilayer Lipid Membrane

**DOI:** 10.1371/journal.pone.0078845

**Published:** 2013-11-12

**Authors:** Chetan Malik, Subhendu Ghosh

**Affiliations:** Department of Biophysics, University of Delhi South Campus, New Delhi, India; University of Cambridge, United Kingdom

## Abstract

Collective behavior of S6 peptide channels derived from KvAP (a bacterial potassium channel) incorporated in lipid bilayer membrane, has been investigated at various applied potentials through multi-channel electrophysiological experiments. The current versus time traces at any particular membrane potential show clear steps for sequential opening of the multi-channels. The minimum current (representing one-channel current) was found out from the amplitude histograms. Accordingly, the number of open channels corresponding to a particular open state was calculated. It was observed that the above-mentioned one channel current is higher than the corresponding single-channel current at most of the applied membrane potentials. Moreover, the difference between the single and one channel conductances is a nonlinear function of the membrane potential. We conclude that the S6 multi-channels show co-operative gating. Voltage relaxation studies support the above-mentioned conclusion.

## Introduction

Ion channels are a well-known group of proteins forming passage across the cell or organelle membranes facilitating transport of ions and metabolites selectively or non-selectively. During the last two decades remarkable progress has taken place in the studies on single-channel electrophysiology. However, in cells ion-channels exist and function in clusters [1], [2]. As a result collective behavior of ion channels has become the key to understanding several phenomena in membrane biology. Collective behavior has been recognized as an important phenomenon in several branches of biology like ecology, cell biology, molecular biology, microbiology, animal and human physiology, behavioral science etc. [3]–[5]. A general observation is that the behavior and function of a system often deviate from that of an individual unit in the system [6]–[8]. One of the reasons for this deviation is the cooperative interaction among the individuals. The clusters of ion channels mentioned above quite often behave cooperatively, thus self-organize and control the ion-flux across the membrane [9]. Consequently, changes in the structure and function of one channel affect the neighboring channels’ activities.

A number of experimental evidences have come up on the co-operativity of various ion channels and its importance over the last few decades. Keleshian *et al* has shown through patch-clamp experiments that coupling of Nicotinic Acetylcholine receptors (two channels) enhances mutual open probability [10]. Clustering and coupled gating in KcsA, a potassium channel from prokaryotes, has been demonstrated in planar lipid bilayers and giant liposomes through electrophysiology experiments [11]. Recently it has been shown that type 2 Ryanodine receptors (RyR2), the cardiac calcium release channels, are closely packed on the sarcoplasmic reticulum membrane and they exhibit simultaneous or coupled gating [12]. Similar phenomenon has been shown by the authors for another Ryanodine receptor (RyR1) from skeletal muscle [13]. The authors argue that coupled gating may be an important regulatory mechanism in excitation-contraction and other Ca^2+^ signaling pathways. Importance of cooperative activation of sodium channels in cortical neurons has been highlighted by Naundorf *et al.* who proposed a new model of action potential [14]. Further it has been shown theoretically that the presence of cooperative channels has a functional impact of enhancing the spike encoding of rapidly varying signals [15]. Simultaneously, theoretical considerations of collective behavior of ion channels have been reported by a number of authors [16], [17] and related models, e.g. Ising and other stochastic models, have been proposed [18]–[21]. The above-mentioned contributions all together highlight the importance of collective behavior of ion channels. In the present work we have investigated the collective behavior of S6 peptide derived from KvAP channel on bilayer lipid membrane (BLM).

KvAP is a potassium channel first isolated from bacterium *Aeropyrum pernix*. It has high sequence similarity with the other voltage dependent potassium channels [22]. Functional KvAP channel is composed of six trans-membrane domains (S1–S6) [23], [24]. Out of six domains, S1–S4 are associated with the movements of the potassium channel within the membrane, while S5 and S6 line the pore region [22], [24]. The voltage sensitivity of the channel arises due to the positively charged amino acids associated with the S4 segment [24]. The activation gate is composed of four S6 segments of pore domains. The former is present near the inner helix bundle and it opens upon activation of the channel [25]–[27]. Regarding the general details of the Potassium channels the crystal structure of the bacterial potassium channel KcsA provides information about the position and the nature of the activation gate [28]. Our aim is to carry on functional studies of the S6 channel-channel interactions, if any, on a bilayer lipid membrane through electrophysiological experiments. Multi-channel current recordings reveal that there exist interactions among S6 channels. In addition, we investigated this through Relaxation studies of S6 channel current.

## Materials and Methods

S6 segment corresponding to voltage dependent potassium channel (KvAP) derived from bacterium *Aeopyrum pernix*, was gifted by Dr. J.K. Ghosh, Central Drug Research Institute, Lucknow, U.P., India, and incorporated in the bilayer membrane as described in [29]. Briefly, the apparatus consisted of a polystyrene cuvette (Warner Instruments) with a thin wall separating two aqueous compartments containing buffer having composition of 500 mM KCl, 5 mM MgCl_2_, and 10 mM HEPES. The pH of the buffer was adjusted to pH 7.4 using Tris-Cl (1 mM). The polystyrene divider had a circular aperture with a diameter of 150 µm. Aqueous compartments were connected to an integrating patch amplifier (Axopatch 200B, Axon Instruments, U.S.A.) through a matched pair of Ag/AgCl electrodes. The cis chamber was connected to the head stage (CV-203BU, Axon Instruments, U.S.A.) of the amplifier, and the trans- chamber was held at virtual ground. Cholesterol solution was prepared by dissolving 25 mg of Cholesterol powder in 1 ml of chloroform (w/v). Cardiolipin concentration was 25 mg per 100 ml of chloroform (w/v). A mixture of Cardiolipin (Avanti-polar Lipids, U.S.A.) and Cholesterol (Avanti Polar Lipids, U.S.A) was prepared in the ratio of 6∶1. The mixture was dried slowly in a stream of nitrogen gas and later 10 µl of *n*-decane (Sigma-Aldrich, U.S.A.) was added. This solution was used over the aperture to form the membrane. The stock solutions of the peptides were made in distilled water to get the desired concentration as mentioned in Verma *et al*, 2011 [29]. Reconstitution of these peptides in BLM was initiated by adding different concentrations of peptide to the BLM chamber to get the recordings (single & multi-channel). The single channel recording was obtained at the concentration of 50 pg of peptide in BLM buffer. The buffer was mixed using a magnetic stirrer. For getting the multichannel recording higher than 50 pg of peptide was used. Channel current was recorded using Digidata (1440A, Axon Instruments, U.S.A.) and the acquisition software CLAMPEX (PCLAMP 10.2., Axon Instruments, U.S.A.). Both single channel and multi-channel current vs. time recordings were performed in a symmetric bath solution (1 ml in both cis and trans chambers) at fixed applied membrane potentials in the range of −100 mV to +100 mV. Sampling frequency of 10 KHz and low pass filter of 1 KHz were used. The temperature of the experimental chamber was maintained at 25°C. Voltage Ramp recording was done from −100 mV to +100 mV at the rate of 20.8 mV/sec.

### Analysis of Electrophysiological Data

Steady state conductance (current/voltage) of the peptide channel was calculated from the single channel current data as well as each open state of the multi-channel current data using the software CLAMPFIT (pCLAMP 10.2, Molecular Devices, CA, U.S.A.). Data were analyzed in Origin 5.0 (Originlab Corp. USA), pCLAMP 10.2 and Axograph X (Molecular Devices, CA, USA).

### Analysis of Histograms

All point Amplitude Histogram was obtained corresponding to each current-time trace using the pClamp software. One minute recording at each voltage was used for histogram analysis. Different states were identified in the histogram and each state was given Gaussian fitting and averages of three different sets of experiments were noted down. Fitting results were represented on the peak. Amplitude histograms help us to identify not only different states but also to find the sub-states if any.

### Calculation of Number of Channels Open in a Multi-Channel Trace

From a multi-channel current recording at a particular membrane potential the all point amplitude histograms were obtained and their mean points were decided. The minimum current difference was identified which was assigned to be the ‘one-channel current’ in the multi-channel ensemble (*Imin*). The number of channels open was calculated by dividing the total current for a particular open state by the ‘one-channel current’. Difference between conductances of single channel (*G_single_*) and one channel (*G_min_*) is defined as follows:
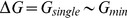
(1)


### Relaxation Analysis

When the voltage is applied to the membrane, it shows sudden jump in the value of current due to sudden instability in the system. This is reflected in the channel current trace. The system reaches from a non-steady state to a steady state and the current changes exponentially. The corresponding time taken in this process (from the point 
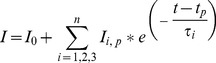
(2)where, it reaches its maximum value to the point where it reaches a stable value) is called relaxation time (τ). This curve was given a triple exponential fitting according to the equation

Here, *I_,i,p_* denotes the peak value of the channel current (positive or negative) at a particular voltage at a time *t = t_p_* (when the relaxation begins). It decays exponentially to a stable value *I*_0_. *I* and *I*_0_ were measured experimentally. τ_1_, τ_2_, τ_3_ were calculated from the equation (2) [30], [31] by simulation. Best fit values (corresponding to least chi square values) were obtained with the help of experimental data using software Origin.

## Results and Discussions

**Figure 1:**
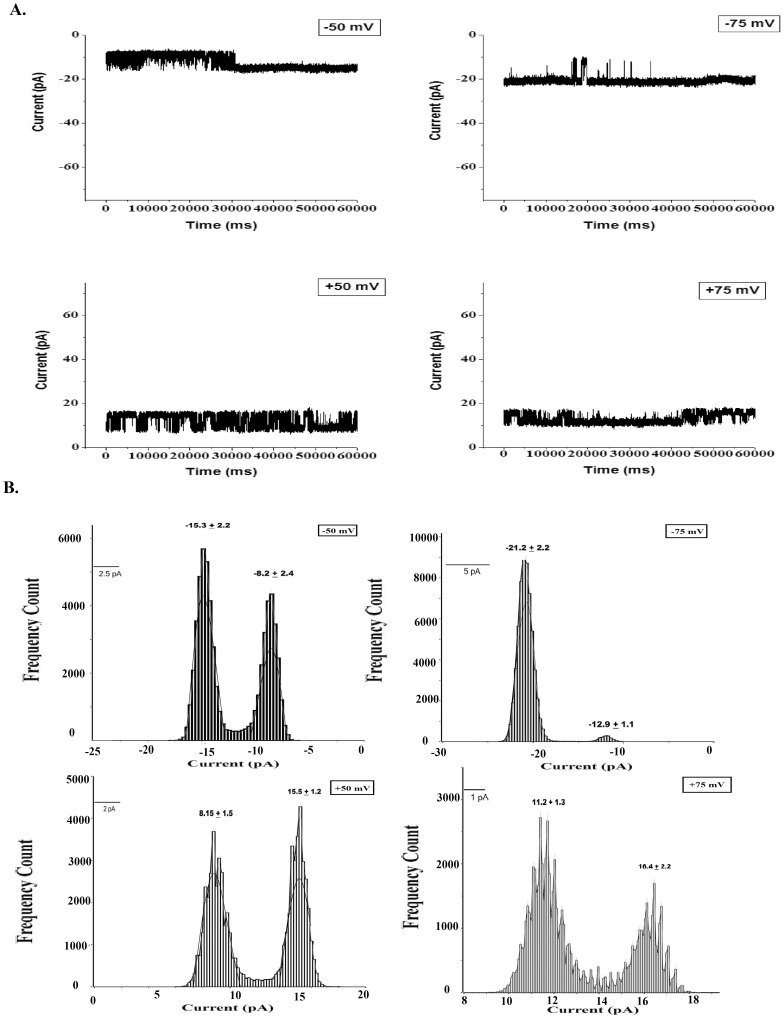
Single channel current traces of S6 incorporated in Cardiolipin BLM. (**A**) Current versus time recorded at applied potential ±50 mV and ±75 mV. In all the recordings baseline was adjusted to 0 pA. (**B**) The amplitude histograms corresponding to A. Different states were given gaussian fitting and the value of the fitting represents Mean±S.D (n = 3).

**Figure 2:**
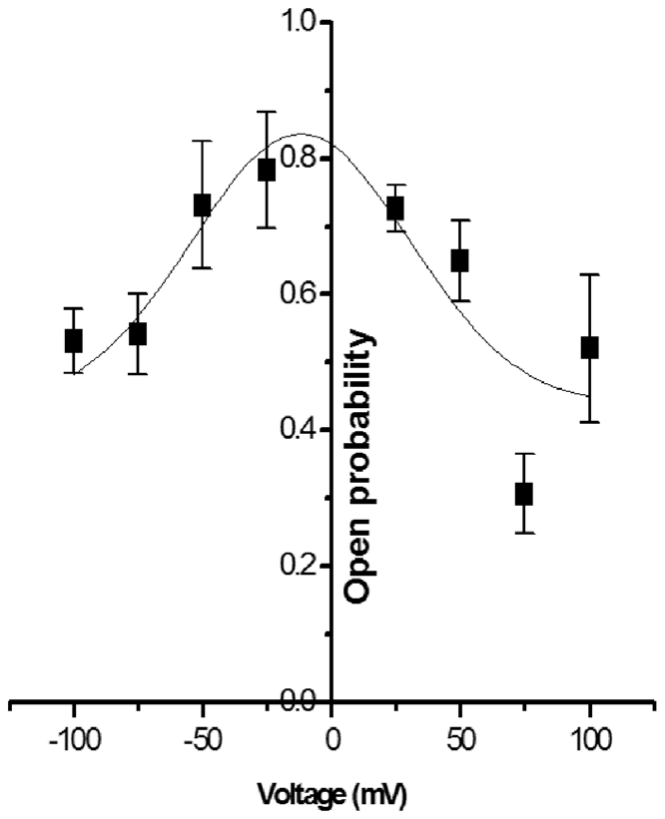
Open probability Distribution of S6 Single channel incorporated in Cardiolipin BLM. The curve shows bell shaped distribution. Each data point represents Mean±S.D. (n = 3).

**Figure 3:**
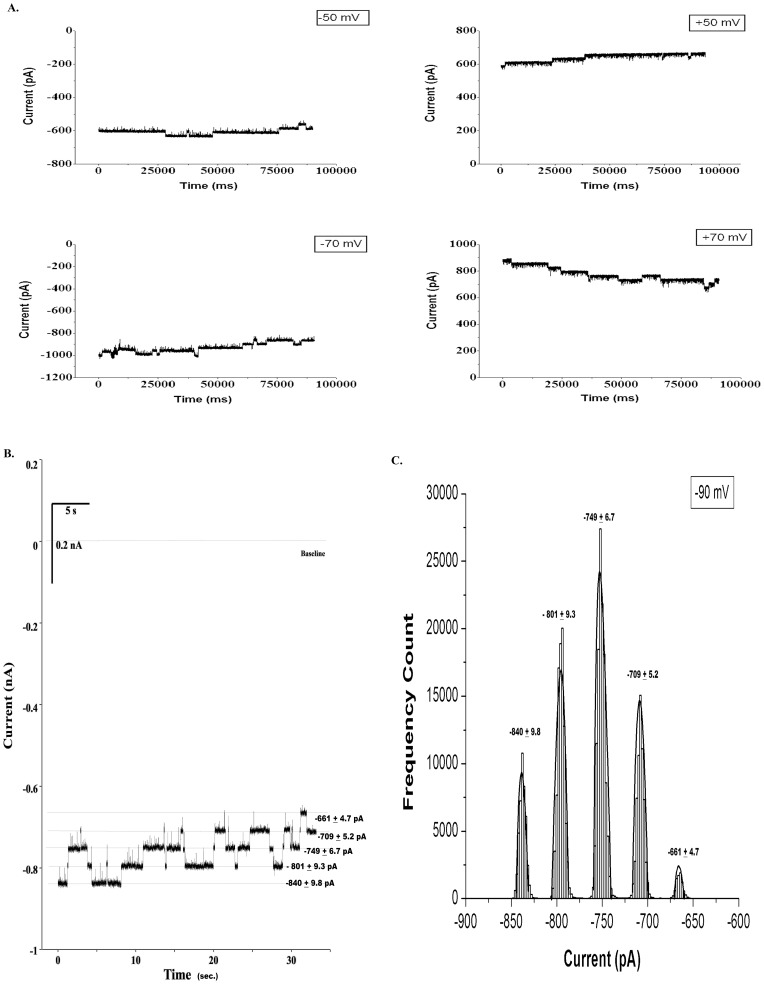
Mutichannel current traces of S6 channel incorporated in cardiolipin BLM. (**A**) Current versus time recorded at applied potential ±50 mV and ±70 mV. Baseline was adjusted to 0 pA in all the recordings. (**B**) Current versus time trace recorded at −90 mV. (C) Amplitude histograms corresponding to (B) showing different open states. The current values obtained by gaussian fitting represent Mean±S.D. (n = 4). Signal was filtered at 1 KHz and sampled at 10 KHz.

**Figure 4:**
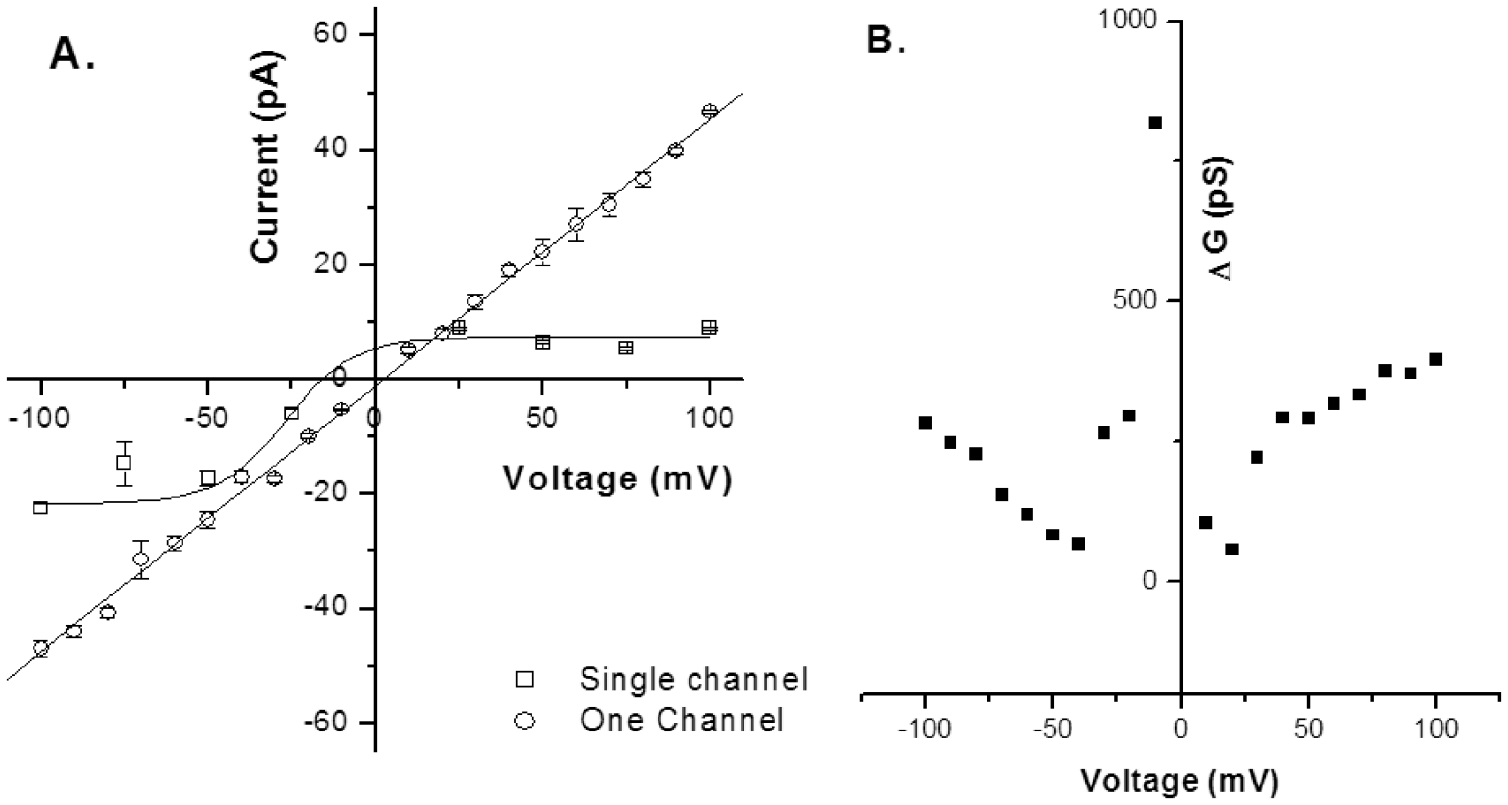
Comparison of Electrophysiological Data of Single and One Channel. **A.** The current versus voltage (I-V) plots of the S6 channel incorporated in cardiolipin BLM. □ Single channel, ○ One channel (calculated from multichannel current as described in Materials & Methods). **B.** Conductance difference (Δ*G*) in pS of Single-channel and One-channel at different applied potentials [derived from A as per Equation (1)]. Number of experiments n = 3 for single channel and n = 4 for multichannel.

**Figure 5:**
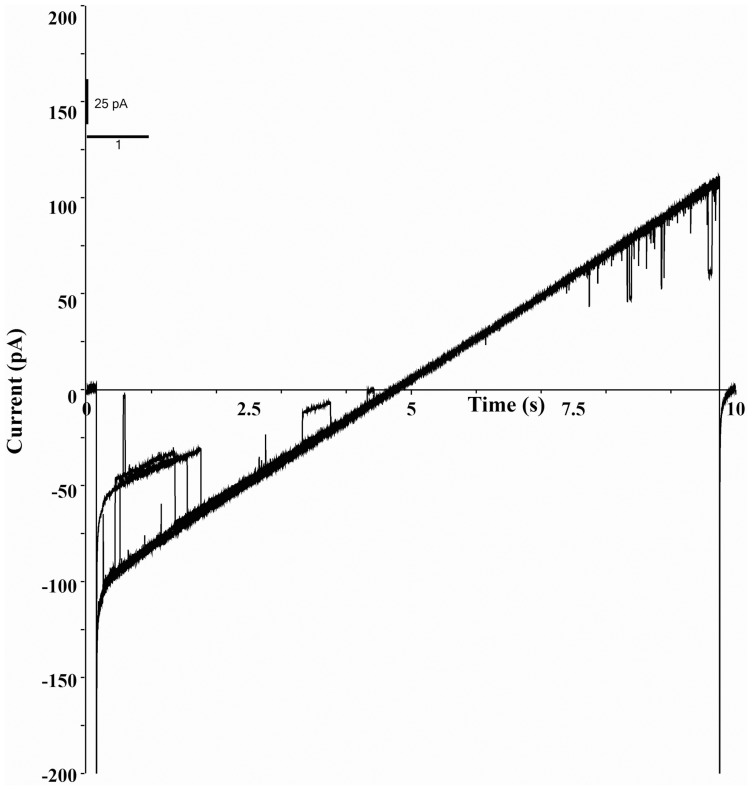
Voltage Ramp of Two Channels. Membrane potential varies from −100 mV to +100 mV at the rate of 20.8 mV/sec. Total trace time is 10 sec. Figure shows five traces (without averaging). Experimental conditions were same as in Fig. 3.

Fig. 1A shows the steady state single-channel current (*Isingle*) vs. time recordings of S6 channel on cardiolipin BLM at ±50 mV and ±75 mV. The traces show clear steps. Here the open and close states are distinctly identifiable. The corresponding amplitude histograms were plotted for each trace (Fig. 1B). As evident from the histograms there are two distinct peaks indicating single opening state and absence of sub-states. Gaussian fitting of the individual peak gives us the mean value of current which has been represented on the individual peak. The nature of the current trace is similar to that of the single-channel S6 in DPhPC BLM [29]. It was observed that single-channel S6 open probability versus voltage plot follows a bell-shaped curve (Fig. 2) with the maximum open probability greater than 0.8, i.e. minimum closed probability of less than 0.2 at 0 mV. However, extensive multi-channel recordings of current versus time show that there is hardly a fully closed state meaning the individual channels in a multi-channel cluster prefer to open together and remain in the open states collectively. This is a good indication of cooperative behavior of the S6 channels. Similar argument has been given by Molina et al in case of two KcsA channels [11]. Fig. 3A shows representative multi-channel current vs. time recordings of S6 channel on cardiolipin BLM at ±50 mV and ±70 mV. As observed in Fig. 3B (−90 mV) the channels open in steps unlike big channels and it is possible to count the number of open states. The corresponding amplitude histograms of −90 mV trace are shown in Fig. 3C, which indicate that different levels (different number of channels) of openings are separately identifiable. For each applied membrane potential the minimum current difference (*Imin*) between a pair of open states was calculated as described in the **Materials & Methods**. This was followed by calculation of the number of channels open corresponding to different open states at the voltage. The S6 channel currents *Isingle* and *Imin* at different voltages (−100 mV to +100 mV) are shown in Fig. 4A. As observed from the figure S6 single-channel undergoes saturation (the current becomes constant) beyond +25 mV and −50 mV applied membrane potentials, hence deviation from Ohm’s law. This means that with increase in the magnitude of the membrane potential the passage through S6 assembly is getting constricted in order to compensate for the higher velocities of the ions across the membrane. However, single-channel (one channel) in the multichannel S6 shows linear I-V plot indicating that no constriction of the passage created by the peptide assembly. The reason could be that in case of multimeric channels S6 single channel can change their cluster organization, e.g. reorganize the cluster size and shape, so that the voltage stress on the channel is undone at least partially. It is clear from Fig. 4A that at most of the membrane potentials *Imin* has higher magnitude than *Isingle*. This shows that due to cluster formation the current per single channel increases. This indicates channel-channel cooperation. The difference of these two conductance values (Δ*G*) as calculated at different voltages indicates that the difference increases with voltage in a nonlinear fashion. Hence the extent of co-operativity depends on the voltage nonlinearly. It may be noted from Fig. 4A that the current amplitudes are not symmetric in positive and negative membrane potentials. This is a phenomenon of rectification of single S6 channel. The reason could be as follows. A part of the channel forming peptide, e.g. side chain of some amino acids, is possibly hindering the flow of ions at positive voltages. However, this property of rectification disappears due to multi-channel cluster organization. A plausible reason has been discussed later in this section. With a view to find out the minimum number of channels required to behave cooperatively, we have recorded two-channel ramp for 10 seconds. Fig. 5 shows the two channel voltage ramp, total 5 recordings, all of which are linear. Hence, it is concluded that two channels are adequate for cooperative behavior. This cooperativity is maintained as the number of channel increases.

**Figure 6:**
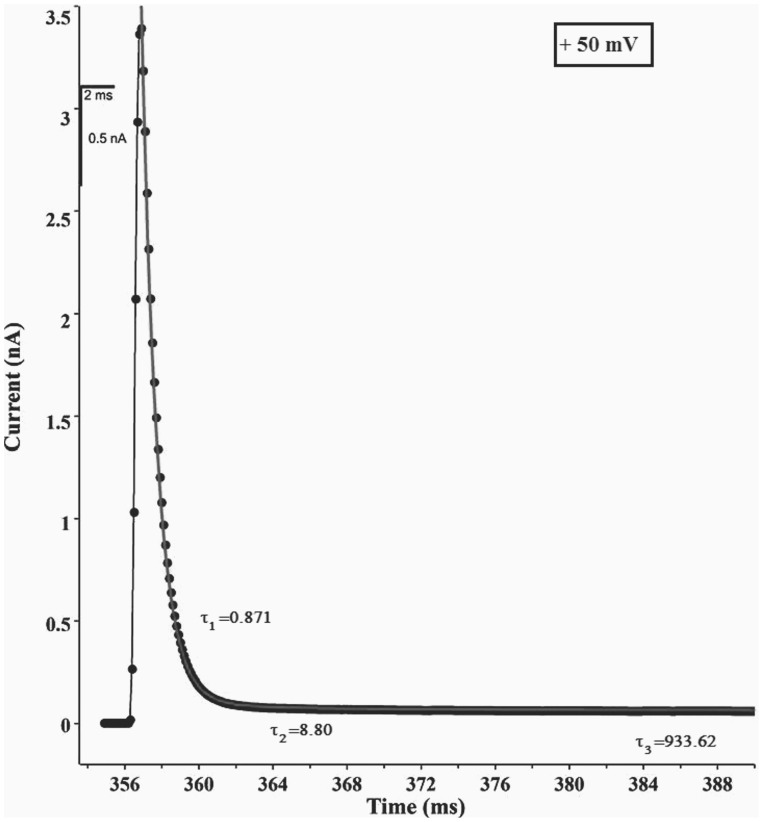
Relaxation of S6 Multi-channel in Cardiolipin BLM. The voltage relaxation curve for +50 mV fitted to three exponentials as described in Materials & Methods (Equation 2). Figure shows the simulated values of relaxation time constants (τ_1_, τ_2_, τ_3_). Experimental conditions were same as in Fig. 3.

**Figure 7:**
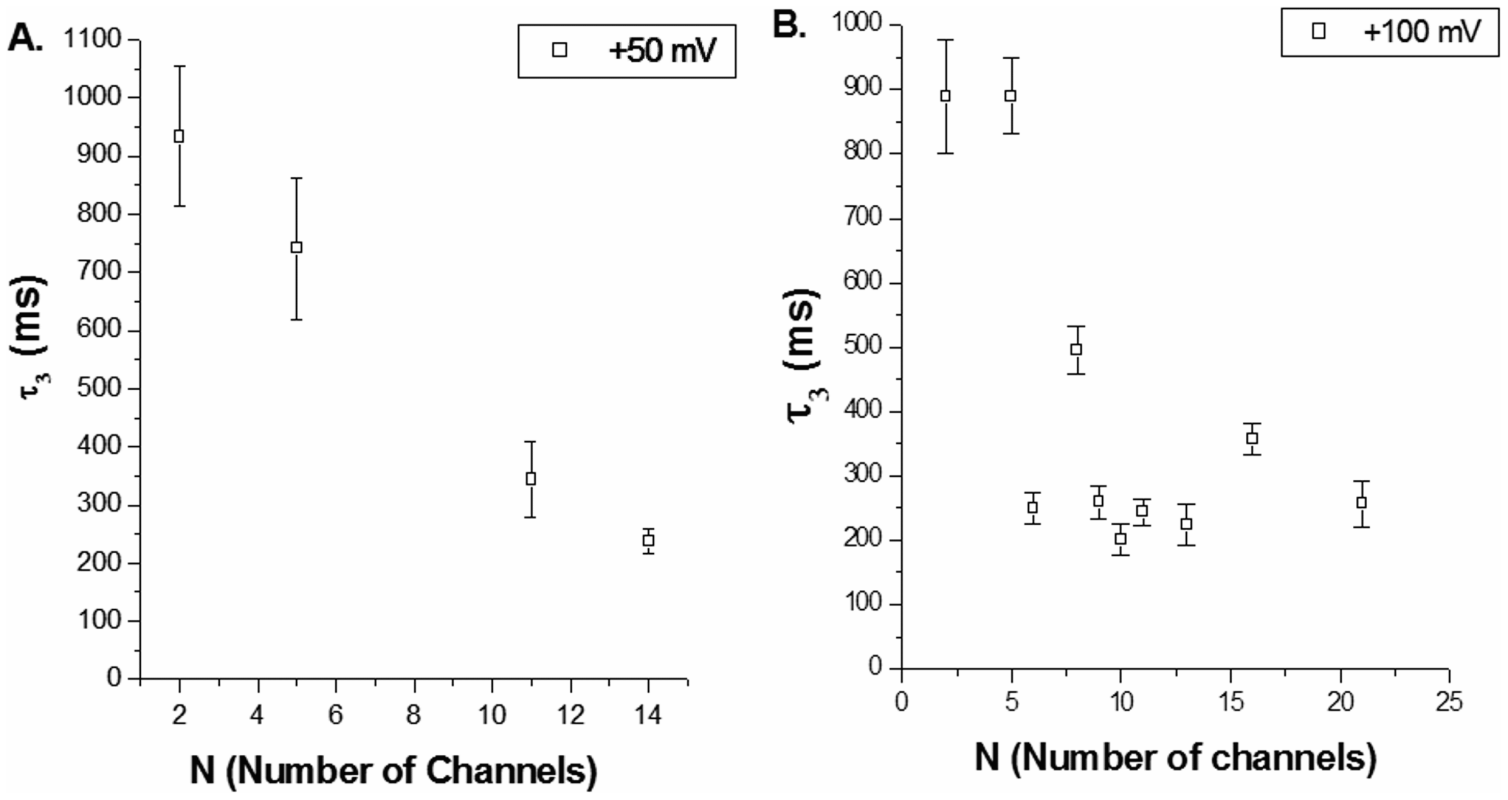
Variation of Relaxation Time Constant (τ_3_) with number of channels. **A.** at membrane potential +50 mV. **B** at membrane potential +100 mV. τ_3_ varies significantly with number of channels inserted. The data shown are average values of 3 experiments (Mean±S.D). Experimental conditions were same as in Fig. 3.

In order to investigate the co-operativity in S6 channels we carried out Relaxation studies as described in **Materials & Methods**. We have already established Relaxation methods in order to understand the co-operativity of ion channels, e.g. synthesized 15-Crown Ether channels and Gap Junctions (Cx32) [30], [31]. As per our analyses we found three exponentials are good fit for the experimental Relaxation data of S6 assembly channels (Fig. 6). Out of these the relaxation time constant τ_1_ lies between 0 and 1 msec., while the value of τ_2_ lies between 1 msec. to 10 msec. and they do not vary significantly with the number of channels. The third relaxation time constant (τ_3_) varies from 100 msec. to 1200 msec., (which is much higher than the other relaxation constants) and it varies with the number of channels (Fig. 7). It may be mentioned here that we have carried out the relaxation studies of Cardiolipin bilayer membrane (without any protein insertion). Here the exponential decay in current amplitude on sudden voltage change is due to membrane capacitance. The relaxation time constant obtained for Cardiolipin BLM is less than 1 msec. We observe that τ_1_ is of the same order of that of the pure BLM, hence could be due to the capacitance contribution. Considering its range of values τ_2_ may be assigned as lipid-S6 peptide interaction. Hence, it is τ_3_ (the largest) that reflects channel-channel interaction. As the relaxation time constant of pure cardiolipin BLM is much less in comparison to the τ_3_ values (10^2^ to 10^3^ msec.) of S6 containing BLM the membrane capacitance contribution in S6 relaxation (channel-channel interaction) has been neglected [30], [31]. Fig. 7A & B show representative plots of number of channels open (N) versus τ_3_ at +100 mV and +50 mV respectively. Our analyses of S6 multi-channel relaxation time at different membrane potentials show variation of τ_3_ with N indicating that there is co-operativity. This supports our previous findings based on conductance analyses.

What is the physical basis of the aforementioned co-operativity in multi-channel assembly of S6? As mentioned in our previous publications opening of one channel would lead to a change in ion flux, which might affect the gating of the neighboring channels [19], [20]. In addition, the S6 residues of one channel can have interactions with those of the other channels. Both these factors could be responsible for the channel-channel cooperativity. As per our understanding the S6 assembly on BLM is not a rigid one. It is thought that the assembly is a multimer in a single-channel. In a multi-channel the S6 residues are likely to exchange their positions owing to the dynamics of the membrane, thus they change the pore size and shape of each individual channel in the whole cluster. In fact, the multi-channel cluster, we believe, is best conceptualized as a dynamic assembly with all the S6 peptides interacting with each other. This dynamics is controlled by the applied membrane potential. The exchange of S6 peptides within a cluster demands lateral movement of the peptides which does not require very high energy. This could be provided by the electrical potential applied across the membrane. Moreover, the time scale of the movement of the peptide backbone is nsec., which is much smaller than the gating time. Hence, during the process of a single gating the peptides can exchange their positions on the membrane (within the cluster of multi-channels) for million times [32]. Right at this moment it is not possible to state how many S6 peptides aggregate in a single channel and a multichannel assembly. But the One channel current (*Imin*) remains the same as observed in our recordings. This suggests that in a multichannel assembly, the number of subunits assembling per channel in the membrane is the same. The fact that rectification property of S6 assembly disappears in multi-channel cluster organization, is plausibly due to the above-mentioned dynamics that keeps enough room for equal passage of anions and cations (this has been already mentioned). If the rectification in single-channel current (*Isingle*) is due to the hindrance caused by a part of the channel forming peptide at higher positive voltages (as mentioned earlier) then it is understood from our results (Fig. 4A) that the movement of that part is restricted or inhibited in the multi-channel assembly on the BLM. The question whether the above-mentioned dynamic stability is thermodynamically favored or not needs to be addressed with detailed analysis, which is our future project. We think the above-mentioned S6 dynamics and inter-peptide interactions is the basis for the channel-channel co-operativity as emerged from our experimental findings.

Our investigation was confined to S6 assemblies and their cooperative nature. Hence, it is not straight forward to apply our findings to KvAP functioning. In a KvAP channel there are four S6 domains placed laterally across the bilayer membrane. It is likely that the interactions among S6 residues or their assemblies are the responsible factors to hold all the S6 domains together and make the KvAP channel functioning. On the bacterial membrane the KvAP channels are expected to form clusters. There S6 tetramers of different KvAP channels might interact cooperatively to boost the total current. However, this could only be confirmed after we obtain the electrophysiology experimental data of KvAP.

Earlier we have reported channel-channel cooperativity for 15 Crown ether assembly and gap junctions (Connexin 32) on BLM. Our present data on S6 assembly channels (current vs. time) has some similarity with 15 Crown ether channel as both the channels (multi) show stepwise opening, the former indicates single-channel steps whereas the latter shows a few channels opening together. On the contrary, the opening steps are bigger for gap junctions showing cluster gating in multi-channels. Also, the extent or degree of cooperativity differs in all the three types of channels as reflected by their respective τ_3_ values.

Based on our studies we may conclude that S6 peptide channels assemble on the lipid bilayer membrane and show co-operativity in multi-channel gating. One of the advantages we observed in this system is that the multi-channel opening is stepwise, which helped us to find the One-channel current in the cluster. This is true with mostly small ion-channels. Hence, the aforesaid approach and method could be used for similar channels.

## Supporting Information

Figure S1 **Relative Conductance (Δ** ***G/Gmax*** **) plot for single-channel and one-channel at different applied membrane potentials derived from** **Fig. 4A** **.** Number of experiments n = 3 for single channel and n = 4 for multichannel.
(TIF)
